# No evidence of oligoclonal bands, intrathecal immunoglobulin synthesis and B cell recruitment in acute ischemic stroke

**DOI:** 10.1371/journal.pone.0283476

**Published:** 2023-03-31

**Authors:** Kornelia Laichinger, Paula Bombach, Jutta Dünschede, Christoph Ruschil, Maria-Ioanna Stefanou, Evelyn Dubois, Sven Poli, Katharina Feil, Ulf Ziemann, Markus Kowarik, Annerose Mengel

**Affiliations:** 1 Department of Neurology & Stroke, and Hertie-Institute for Clinical Brain Research, Eberhard-Karls University of Tübingen, Tübingen, Germany; 2 Interdisciplinary Division of Neuro-Oncology, Center for Neuro-Oncology, Comprehensive Cancer Center Tübingen Stuttgart, University Hospital Tübingen, Eberhard-Karls University Tübingen, Tübingen, Germany; Policlinico Riuniti of Foggia: Neuroscience Department, S.C. Ospedaliera of Neurology-Stroke Unit, ITALY

## Abstract

**Background:**

Within the past 10 years, immune mechanisms associated with acute ischemic stroke (AIS) have been brought into focus, but data on B cell activation and intrathecal Ig production is still scarce. In this study, we determined the prevalence of an elevated IgG index, positive oligoclonal bands (OCBs) and chemokine C-X-C motif ligand 13 (CXCL13) levels in the cerebrospinal fluid (CSF) as markers of intrathecal IgG synthesis and B cell activation in patients with AIS.

**Methods:**

In a retrospective study we analyzed the cerebrospinal fluid (CSF) from 212 patients with AIS from December 2013 to May 2018 assessing intrathecal Ig synthesis, OCBs and CXCL13 concentrations.

**Results:**

Overall, 5.7% (12/212) of AIS patients showed an intrathecal IgG synthesis, 0.5% (1/212) with isolated elevated IgG index, 5.2% (7/136) isolated positive OCBs and 2.9% (4/136) both elevated IgG index and positive OCBs. CXCL13 levels were elevated in 3.6% (3/83) of the patients. Approximately one third of these patients had simultaneously chronic inflammatory CNS disease (multiple sclerosis, neuromyelitis optica spectrum disorder, neurosarcoidosis). There was no significant association between CSF findings and stroke characteristics including vascular territory, localization, volume, etiology, acute treatment, or blood-brain barrier dysfunction. Intrathecal IgG synthesis was more common in patients with prior stroke. Longitudinal CSF analysis did not reveal any newly-occurring, but instead mostly persistent or even disappearing intrathecal IgG synthesis after AIS.

**Conclusions:**

We found no evidence of a relevant B cell recruitment and intrathecal IgG synthesis in patients with AIS. In fact, the occurrence of intrathecal IgG synthesis was associated with concurrent chronic inflammatory CNS disease or previous stroke. Consequently, in patients with first-ever AIS and intrathecal IgG synthesis, physicians should search for concomitant inflammatory CNS disease.

## Background

During the last years there has been an increasing focus on immune mechanisms associated with acute ischemic stroke (AIS). Meanwhile it is known that damage associated molecular patterns (DAMPs) including chemokines and cytokines that are mainly secreted by innate immune cells are associated with a dysfunction of the blood-brain barrier (BBB) leading to an influx of leukocytes into the central nervous system (CNS). Studies have shown that neutrophils are the first immune cells that invade the brain whereas lymphocytes dominate the later immune responses [1, 2]. Regarding B cells, their role during stroke has not been fully elucidated and only limited data is available as of yet.

Early studies reported an intrathecal immunoglobulin (Ig) synthesis and CSF specific oligoclonal bands (OCBs) in the cerebrospinal fluid (CSF) of stroke patients [3–5]. More recently, Prüss and colleagues examined the CSF of 318 patients with acute “non-inflammatory” stroke with 24.8% of the patients showing an intrathecal Ig synthesis and 17.9% CSF specific OCBs. Furthermore, 31.3% of the stroke patients displayed a BBB dysfunction. A fraction of the tested patients underwent a second lumbar puncture at follow-up and 50% of them developed intrathecal IgG synthesis. In this study, OCBs positive patients were neither significantly different in age, sex, type of ischemia, frequency of pleocytosis, BBB dysfunction nor in the existence of relevant previous illness or current system infection compared to their OCBs negative counterparts. There was also no significant difference in the presence of OCBs in patients with first-ever stroke versus patients with old infarct before the index one [5]. By contrast, Bolayir and colleagues (2014) showed in a cohort of 51 patients with acute stroke that the CSF IgG level, the CSF IgG/albumin ratio as well as the CSF IgG index were significantly higher in patients with recurrent stroke. In patients with repeated stroke there was also a significant positive correlation between the infarct volume and the level of CSF IgG [6]. These data point towards a substantial intrathecal Ig production in stroke and raise the question what pathophysiological roles B cells may play during stroke-specific immune reactions. Other studies have shown that B lymphocyte infiltration into the brain starts approximately 7 days post-stroke and is maintained for at least 10 to 12 weeks [7, 8]. During the acute phase of stroke, interleukin-10 (IL-10) producing B cells together with T reg cells can dampen the inflammatory process and reduce infarct lesion size due to a reduced recruitment of neutrophils [7, 9–11]. On the other hand, neuronal damage and BBB dysfunction lead to the exposure of neuronal antigens that are likely to be exposed to B cells. Moreover, there is experimental evidence of a specific antigen-dependent T cell and B cell interaction as well as findings of antibody secreting plasma cells around the infarct lesion [7, 8]. This evidence has fueled the hypothesis that there might be a development of CNS specific autoantibodies after stroke in some patients [7, 12, 13]. In contrast to the aforementioned beneficial effect of B cells post-stroke, this delayed B cell activation possibly leads to the development of cognitive deficits that extends from cognitive decline to post-stroke dementia [8]. Thus, it seems that B cells may play a beneficial or conversely a detrimental role after stroke under specific circumstances.

Due to the surprisingly high incidence of OCBs in former studies, especially in the study by Prüss et al. with nearly one quarter of stroke patients showing an intrathecal IgG synthesis, we aim to evaluate the occurrence of an intrathecal IgG production and OCBs in an AIS patient collective. Since CSF CXCL13 levels have strongly been associated with the elevated fraction of B cells into the CSF [14, 15], we also measured CSF CXCL13 concentrations to monitor intrathecal B cell recruitment during AIS.

## Methods

### Patients and study design

In a retrospective study design, patients with AIS who were hospitalized at the Department of Neurology, University of Tübingen, Germany from December 2013 to May 2018 were evaluated. The study was conducted in accordance with the Declaration of Helsinki and was approved by the ethics committee of University Hospital Tübingen (protocol number 329/2019BO1). All Patients underwent cerebral imaging by computer tomography (CT) or magnetic resonance imaging (MRI). Cerebral ischemia was diagnosed either radiologically or clinically (symptoms > 24 hours). 212 patients underwent lumbar puncture including determination of IgG index during their routine clinical work up for AIS. Indications for lumbar puncture comprised elevated inflammatory parameters in the absence of concomitant infection, suspected vasculitis or CNS infection, seizure, cognitive impairment, persistent confusion, reduced vigilance or diagnostic uncertainty regarding stroke etiology.

### Data collection

Intrathecal Immunoglobulin Synthesis was defined as either elevated IgG index or positive OCBs. The IgG index was calculated from the ratio of IgG CSF/serum quotient and albumin CSF/serum quotient and was defined as elevated with a ratio higher than 0.7. The amount of IgG and albumin in CSF and serum samples was measured by nephelometry (analyzer used: BN ProSpec System from Siemens Healthineers). CSF specific OCBs were mostly (n = 99) determined by isoelectric focusing followed by silver staining, only in a few samples (n = 37) that were measured later an immunoblot was carried out (gel used for both methods: polyacrylamidgel, exactly IEFGel 6–11 24S from edc Tübingen). Conventionally, OCB type 2 (OCBs in CSF only) and 3 (OCBs in CSF and serum, with additional OCBs in CSF) were defined positive [16]. Blood-CSF barrier (BCB) dysfunction was estimated by albumin CSF/serum quotient. CXCL13 concentrations were measured by ELISA test (R&D, DY801, DuoSet ELISA) according to the manufacturer instructions. We could measure CXCL13 levels in 83 of our AIS samples and values above the cut-off level of 8,6 pg/ml were defined as elevated. The measurements also included positive samples (N = 5) and negative controls (n = 5), previously measured during the routine diagnostic work up of patients. All measurements were within the expected concentration range, longitudinal measurements of the same samples did not differ more than 10%. In general, the CSF was usually freshly analyzed, cryopreserved material was only used for the re-determined samples.

Stroke localization (cortical, subcortical, infratentorial), side (right, left, both) and vascular territory (anterior and/or posterior circulation) were determined mainly on basis of cerebral imaging using either cerebral CT or if available MRI. Infarct volume was calculated with ABC/2 and summed up in case of multiple lesions, on the basis of cerebral CT or if available MRI using the diffusion weighted or T2 FLAIR sequences.

Infarct etiology was assessed by cardiovascular evaluation classified according to TOAST criteria [17].

### Statistics

Continuous distributed data are presented with median values together with their corresponding interquartile range (IQR) and counts with percentages for all categorial or dichotomous variables. Differences between patient groups with elevated versus normal IgG index were assessed with Pearson’s chi squared test using SPSS version 28. Results were considered as statistically significant for a two-sided p-value of 0.05 adjusted by Bonferroni correction in case of multiple testing.

## Result

**Fig 1:**
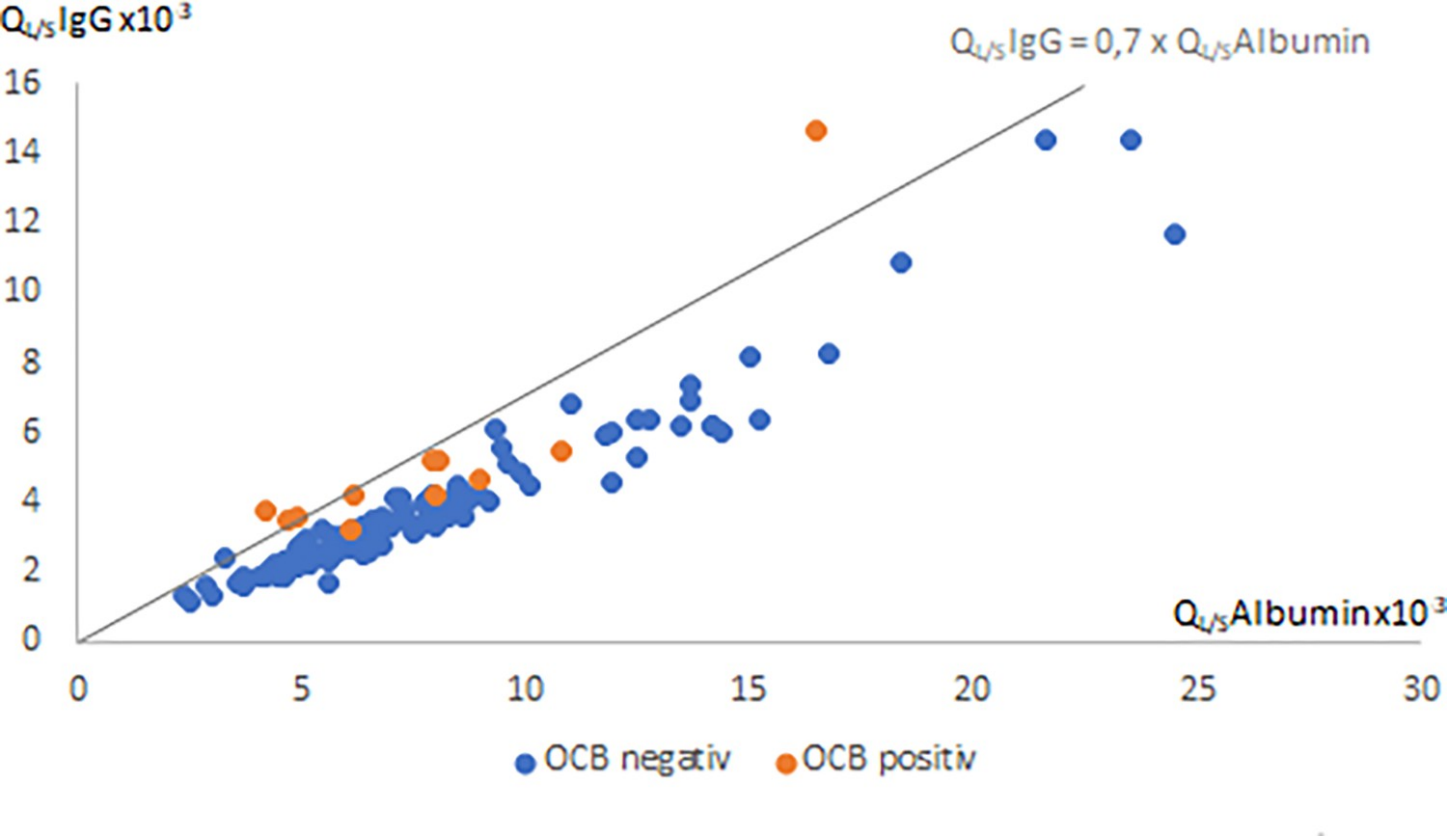
Quotient of CSF and serum IgG as well as quotient of CSF and serum albumin for all OCB positive (red spots) and OCB negative (blue spots) patients.

212 Patients with AIS underwent lumbar puncture including the analysis of IgG index, and in 136 of them OCBs. In addition, CSF CXCL13 concentrations were measured in 83 out of 212 patients. When looking at our data set, 5.7% (12/212) of the patients showed an intrathecal immunoglobulin synthesis; 0.5% (1/212) had an elevated IgG index only, 5.2% (7/136) positive OCBs only and 2.9% (4/136) showed both, an elevated IgG index as well as positive OCBs. Fig 1 shows the status of OCBs and IgG index for each patient.

CXCL13 concentrations were elevated in 3.6% (3/83) of the patients with two patients showing positive OCBs and one patient showing OCBs type 4. Thereby the exact values were 54.33 pg/ml in a patient with assumed vasculitis, 12.66 pg/ml in a patient with known multiple sclerosis and 460.09 pg/ml in a patient with neurosarcoidosis.

Patients with and without intrathecal IgG synthesis are compared in Table 1. They were not significantly different in age and sex, although there was a slight tendency of females presenting more frequently intrathecal IgG syntheses. The median latency– 6 days (IQR: 1.25; 8 days) in patients with and 5 days (IQR: 2; 10 days) in patients without intrathecal IgG synthesis–from stroke to lumbar puncture was almost the same in both groups. There was also no significant difference in the occurrence of BCB dysfunction or pleocytosis, although it appears that patients with intrathecal IgG synthesis are more likely to present with mild pleocytosis (5–50 cells/μl). Furthermore, patients with intrathecal IgG synthesis did not differ in any of the stroke characteristics, like vascular territory, side of infarction, infarct volume, localization or performed revascularization treatment (thrombolysis or interventional recanalization). There was especially no difference in relation to suspected etiology and in particular no patient with intrathecal IgG synthesis had stroke due to vasculitis. However, there was a significant difference regarding the occurrence of stroke events, with an elevated IgG index more often detectable in patients with prior cerebral ischemia. There was a significant association between intrathecal IgG synthesis / elevated CXCL13 concentrations and the presence of a concurrent disease. In detail, 33.3% (4/12) of the patients with an intrathecal IgG synthesis had chronic inflammatory CNS disease, that was already known at the time of the current AIS. As illustrated in Table 2, two patients had multiple sclerosis, both with an elevated IgG index and OCB type 3, another patient had neuromyelitis optica spectrum disorder with positive anti-aquaporin 4 antibodies and OCB type 3, but normal IgG index. The fourth patient had isolated neurosarcoidosis (diagnosed by biopsy) with an elevated IgG index and OCB type 2. No patient without intrathecal IgG synthesis had a simultaneous chronic inflammatory CNS disease. 8.3% (1/12) of the patients with intrathecal IgG synthesis had rheumatic comorbidity, in sense of an undifferentiated connective tissue disease with OCB type 3 and normal IgG index. Only 3.5% (7/200) of patients without intrathecal IgG synthesis had a concurrent rheumatic disease. Apart from patients with chronic inflammatory CNS disease, only 3.8% (8/208) of the remaining collective and apart from patients with either chronic inflammatory CNS disease or rheumatic disease, only 3.5% (7/200) of the remaining collective showed intrathecal IgG synthesis. In Table 3, intrathecal IgG synthesis is broken down in more detail according to the presence of positive OCBs and elevated IgG index. All patients with elevated CSF CXCL13 levels showed a concurrent inflammatory disease (one patient each with multiple sclerosis, sarcoidosis and rheumatic disease) and elevated CXCL13 levels were significantly associated with intrathecal IgG synthesis.

**Table 1:** Comparison of patients with and without elevated IgG index.

	Intrathecal IgG Synthesis	No intrathecal IgG Synthesis	P-value	Missing value
Age	median 59 years (IQR = 40)	median 58.5 years (IQR = 25)	0.563	-
Sex			0.059	-
• ♂	3/12 = 25%	106/200 = 53%		
• ♀	9/12 = 75%	94/200 = 47%		
Latency to LP	median 6 days	median 5 days	0.438	1
	(IQR = 1.25; 8)	(IQR = 2; 10)		
Side			0.582	-
• right	4/12 = 33.3%	90/200 = 45%		
• left	7/12 = 58.3%	86/200 = 43%		
• both	1/12 = 8.3%	24/200 = 12%		
Territory			0.958	-
• media/ anterior	7/12 = 58.3%	110/200 = 55%		
• posterior/ vertebrobasilar	4/12 = 33.3%	75/200 = 37.5%		
• both	1/12 = 8.3%	15/200 = 7.5%		
Localization			0.488	3
• cortical	4/11 = 36.4%	63/198 = 31.8%		
• subcortical	2/11 = 18.2%	66/198 = 33.3%		
• infratentorial	4/11 = 36.4%	52/198 = 26.3%		
• multiple with cortical	0	12/198 = 6.1%		
• multiple without cortical	1/11 = 9.1%	5/198 = 2.5%		
Volume			0.526	36
• <21ml	10/10 = 100%	147/166 = 88.6%		
• 21-50ml	0	11/166 = 6.6%		
• >50ml	0	8/166 = 4.8%		
Etiology/ TOAST			0.375	-
• vasculitic	0	8/200 = 4%		
• macroangio-pathic/1	2/12 = 16.7%	20/200 = 10%		
• cardio-embolic/2	2/12 = 16.7%	20/200 = 10%		
• microangio-pathic/3	2/12 = 16.7%	45/200 = 22.5%		
• other cause/4	5/12 = 41.7%	45/200 = 22.5%		
• unclear incl. ESUS/5	1/12 = 8.3%	62/200 = 31%		
Revascularization treatment			0.887	2
• yes	2/12 = 16.7%	30/198 = 15.2%		
First-ever stroke			*0.003	-
• yes	6/12 = 50%	168/200 = 84%		
BCB dysfunction			0.286	0
• yes	3/12 = 25%	81/200 = 40.5%		
Pleocytosis			0.288	
• mild (5–50 cells/ μl)	4/12 = 33.3%	32/200 = 16%		
• marked (>50 cells/μl)	0	2/200 = 1%		
• no	8/12 = 66.6%	166/200 = 83%		
Elevated CXCL13 levels	2/3 = 66.6%	1/80 = 1.2%		129
Concurrent disease			*0.001	-
• chronic-inflammatory CNS disease	4/12 = 33.3%	-		
• rheumatic disease	1/12 = 8.3%	7/200 = 3.5%		
• other/none	7/12 = 58.3%	193/200 = 96.5%		

Abbreviations: IgG = immunoglobulin G, LP = lumbar puncture, ESUS = embolic stroke of undetermined source, BCB = blood-CSF barrier, CNS = central nervous system, * denotes significance. Statistics: Differences were assessed using Persons Chi-Squared test and results were considered as statistically significant for a two-sides p-value <0,05 adjusted to Bonferroni correction.

**Table 2:** Patients with intrathecal IgG synthesis and chronic inflammatory CNS disease.

	Diagnosis	IgG index	OCBs	CXCL13
• Additional chronic inflammatory CNS disease	multiple sclerosis	elevated	type 3	elevated
	multiple sclerosis	elevated	type 3	normal
	neuromyelitis optica spectrum disorder	normal	type 3	normal
	Neurosarcoidosis	elevated	type 2	elevated

Abbreviations: IgG = immunoglobulin G, OCBs = oligoclonal bands, CNS = central nervous system, CXCL13 = Chemokine C-X-C motif ligand 13.

**Table 3:** Occurrence of intrathecal IgG synthesis in context of autoimmune disease.

	intrathecal IgG synthesis	OCBs positive only	OCBs positive + elevated IgG index	elevated IgG index only
• entire collective	5.7% (12/212)	5.2% (7/136)	2.9% (4/136)	0.5% (1/212)
• patients without chronic inflammatory CNS disease	3.8% (8/208)	3.0% (4/132)	0.8% (1/132)	0.5% (1/208)
• patients without chronic inflammatory CNS disease or rheumatic disease	3.5% (7/200)	2.4% (3/124)	0.8% (1/124)	0.5% (1/200)

Abbreviations: IgG = immunoglobulin G, OCBs = oligoclonal bands, CNS = central nervous system.

Longitudinal CSF analysis by a second LP in 22 patients, at least 10 days after the first (median: 302 days) and third LP in 4 patients, at least 10 days (median: 372 days) after the second, revealed no patient with a newly occurring elevated IgG index. Among these patients, two showed an initially elevated IgG index. One of them was diagnosed with multiple sclerosis and showed a persisting elevated IgG index during the second and third analysis besides the OCB type 3 in the initial CSF analysis. The other one already showed normalized IgG index at the second CSF analysis. This patient was diagnosed with isolated neurosarcoidosis and had OCB type 2 in the initial analysis.

## Discussion

The CSF of 212 patients with ischemic stroke was analysed for intrathecal Ig synthesis by determining the CSF specific IgG index / OCBs and CXCL13 concentrations as markers of CNS specific B cell activation. Intrathecal IgG synthesis was present in 5.7% (12/212) of the patients, with 0.5% (1/212) showing an elevated IgG index only, 5.2% (7/136) showing positive OCBs only and 2.9% (4/136) showing both, an elevated IgG index as well as positive OCBs. Regarding CXCL13 concentrations, 3.6% (3/83) of the patients showed elevated CXCL13 levels, however, all of these patients had additional inflammatory comorbidities. One third of the patients (4/12) with an intrathecal IgG synthesis were additionally diagnosed with chronic inflammatory CNS diseases—most likely causal for this finding -, while the remaining two thirds of our patient collective (8/12) showed an intrathecal Ig synthesis without inflammatory diseases. Altogether, the relatively low prevalence of intrathecal Ig synthesis highly contrasts with previous studies.

In a previous work, a substantial intrathecal antibody synthesis of all classes (IgG, IgM, IgA) and positive OCBs, partially also paired with elevated IgG index, were detected in 24.8% and 17.9% or more of patients with AIS, respectively [5]. Of note, in that study patients with known CNS infection, demyelinating disease, head trauma or cerebral neoplasm were excluded [5]. If we likewise exclude patients with chronic inflammatory CNS disease, the percentage of AIS patients with intrathecal IgG synthesis continues to shrink to 3.8% which is more than a factor seven less than previously reported. In addition, it should be emphasized that our technical methods did not differ significantly compared to those in the just referred study by Prüss et al. [5]. The amount of IgG and albumin in CSF and serum samples was measured by routine nephelometry and OCBs were mostly determined by isoelectric focusing followed by silver staining in both studies. One difference to previous studies might be that the time between stroke and lumbar puncture was around 5d in our study but shorter in previous studies (e.g., time window of 96h) [5]. Given that germinal center (like) reactions with the induction of antigen-specific B cells and antibody production is assumed to take at least 7-10d to develop [6], it further seems unlikely that a substantial intrathecal Ig synthesis might occur 96h after an acute event. In line with this hypothesis, some of the patients in the collective of Prüss et al. underwent a second lumbar puncture, and 50% of these patients then showed newly-occurring intrathecal IgG synthesis [5]. However, in our collective longitudinal CSF analysis in a subset of patients didn’t result in new occurring, but at most in persisting or even disappearing intrathecal IgG synthesis.

While Prüss et al. found no significant difference in the occurrence of OCBs with respect to first event of stroke versus recurrent stroke [5], Bolayir et al. observed that the CSF IgG level, the CSF IgG/Albumin ratio as well as the CSF IgG index were significantly higher in patients with recurrent stroke [6]. The latter observation could possibly be explained by the fact that immune cells are recruited to the CNS and sensitized to neuronal antigens following the first stroke and then, after the second hit, intrathecal antibody production by activation of antigen-experienced B cells might occur more quickly [13]. Also, in our collective, intrathecal IgG synthesis was significantly more common in patients with recurrent stroke but still very rare. From a mechanistic point of view, our data do not support the hypothesis of a long lasting, possibly compartmentalized B cell reaction within the CNS compartment following stroke. However, the sole CSF analysis, as performed in our analysis, is not sufficient to definitely rule out a CNS B cell reaction after AIS. Further investigations including histological analyses are therefore necessary.

Furthermore, we must keep in mind that the collective of our retrospective study might be biased. Because of the invasive nature of lumbar puncture, mostly younger patients with unclear stroke etiology or suspected vasculitis were examined, which is reflected by the median patient age of about 59 years. Thus, the classical stroke patient with obvious macroangiopathic, microangiopathic or cardioembolic genesis might be underrepresented in our patient collective. Altogether, this biased patient selection might also contribute to the significant differences between our and previous studies. The variability regarding the duration between stroke and LP is another limitation of our study. In case of a very short latency, the B cell response might not yet be sufficiently developed and in case of a very long latency signs of a passed B cell response could no longer be detectable. On the other hand, it could also be advantageous that data are available for a wide variety of latencies leaving a certain leeway for a possible variable ongoing B cell response. In addition, the evaluation of CXCL13 is limited due to the restricted sample size, but still provides a gain in information in combination with the entire data set, as a further marker of B cell recruitment. As another limitation of our retrospective study, we could not assess all clinically or stroke-relevant parameters for all included patients, however, our collective with a large sample size of included AIS patients seems representative for the purposes of our analyses.

## Conclusion

Taken together, our data do not provide evidence for a relevant B cell recruitment and intrathecal IgG synthesis following cerebral ischemia alone. In our patient collective, the occurrence of intrathecal IgG synthesis was associated with a concurrent chronic inflammatory CNS disease. Consequently, in patients with a first-ever stroke combined with intrathecal IgG synthesis, one should think outside of the box and also search for other underlying diseases such as a chronic inflammatory CNS disorder.

## Abbreviations

AIS: Acute ischemic stroke
BBB: Blood-brain barrier
BCB: Blood-CSF barrier
CNS: Central nervous system
CSF: Cerebrospinal fluid
CT: Computer tomography
CXCL13: Chemokine C-X-C motif ligand 13
DAMPs: Damage associated molecular patterns
ESUS: Embolic Stroke of undetermined source
IgG: Immunoglobulin G
Il: Interleukin
IQR: Interquartile Range
LP: Lumbar puncture
MRI: Magnetic resonance imaging
OCBs: Oligoclonal bands
